# CO- and H_2_S-adsorbed one-dimensional AlSi structures for gas sensing applications

**DOI:** 10.1098/rsos.240774

**Published:** 2025-01-08

**Authors:** Hoang Van Ngoc

**Affiliations:** ^1^ Institute of Southeast Vietnamese Studies, Thu Dau Mot University, Thu Dau Mot, Binh Duong, Vietnam

**Keywords:** adsorption, CO, H_2_S, one-dimensional AlSi monolayers, sensing applications, configurations

## Abstract

The potential applications of low-dimensional materials continue to inspire significant interest among researchers worldwide. This study investigates the properties of one-dimensional AlSi monolayers, specifically AlSi nanoribbons, and their adsorption behaviour with CO and H_2_S molecules. The electronic, magnetic and optical properties of these systems are calculated using density functional theory and the Vienna Ab initio Simulation Package. Results indicate that the structures remain relatively planar with negligible buckling heights. All three studied structures exhibit non-zero magnetic moments; notably, CO adsorption enhances the magnetic moment of the pristine AlSi nanoribbon, whereas H_2_S adsorption reduces it. Adsorption energy calculations reveal that CO exhibits stronger adsorption compared to H_2_S. Furthermore, a detailed investigation of the optical properties—including the real and imaginary parts of the dielectric function, absorption coefficient and electron–hole density—demonstrates the potential of these structures in nanotechnology applications, particularly for CO and H_2_S gas sensing.

## 1. Introduction

Materials science continually demands the exploration and discovery of novel materials with enhanced properties. Among these, low-dimensional materials—specifically two-dimensional (2D) materials [1–3], one-dimensional (1D) materials [4–6] and zero-dimensional (0D) materials [7–9]—have garnered significant attention due to their nanoscale dimensions and the distinct physical properties associated with their reduced dimensionality. These materials exhibit unique characteristics primarily driven by electron confinement, leading to the emergence of a range of novel phenomena. Among these materials, those characterized by hexagonal honeycomb structures, notably graphene [10,11], silicene [12–14], germanene [15–17] and borophene [18–20], command special interest by virtue of their distinctive properties. The quest for graphene analogues across various applications has engendered a profusion of discoveries within the realm of materials science. Silicene, distinguished by its non-planar hexagonal lattice, evinces a buckling phenomenon owing to sp^3^ bonding between silicon atoms [14]. Facilitated by the predisposition of silicon atoms towards sp^3^ hybridization, hydrogenation of silicene is considerably facile in comparison to graphene [14]. Furthermore, the heightened efficacy of spin–orbit interactions in silicene engenders a discernible gap of approximately 1.55 meV at the Dirac point [21]. Molecular dynamics simulations conducted by Roman & Cranford delineate the elastic stiffness of silicene in zigzag and armchair directions to be 50.44 and 62.31 N m^−1^, respectively [22]. Moreover, the study posits the bending stiffness per unit width of silicene to be 38.63 eV, thus affirming the amplification of bending stiffness vis-à-vis graphene owing to its buckled structure. Further investigations elucidate alterations in the electromagnetic properties of silicene upon surface functionalization. Complete functionalization with hydrogen or bromine engenders a non-magnetic semiconducting structure, whereas partial functionalization induces a ferromagnetic or semimetallic semiconducting configuration [23]. Density functional theory (DFT)-based analyses of silicene functionalization underscore the modulation of bandgap contingent upon the nature of the functionalizing atomic groups. Functionalized groups such as Cl–Si–Br, Cl–Si–Cl and F–Si–F are demonstrated to effectuate bandgap variations within the range of 1.25–2.13 eV, while metallic structures ensue with groups like CH–Si–CH and NH–Si–NH [24].

Furthermore, the introduction of dopants into silicene engenders novel structures replete with diverse properties [25–27]. Spin-dependent density functional calculations by Abdullah *et al*. reveal that doping with transition metals attenuates the buckling height of silicene through interactions with silicon atoms, concurrently inducing a marked enhancement in system magnetism [28]. Explorations into the utilization of silicene for NO and CO gas sensing applications evince significantly augmented adsorption sensitivity upon aluminium doping, thereby offering prospects for bandgap modulation or metallization contingent on doping configurations [29]. Beyond doping and functionalization strategies, the application of an external electric field emerges as a viable avenue for tailoring silicene properties [30–32]. Notably, the imposition of a vertical electric field facilitates bandgap modulation in silicene, with gap width exhibiting linear augmentation commensurate with electric field intensity [33].

The transport properties of silicene and its nano-derivatives have been extensively investigated by Ghosal’s research group [34]. In that work, the thermoelectric properties and methods to enhance thermoelectric performance in silicene were also explored. By integrating theoretical analysis with evaluations of potential applications, the team proposed promising uses for silicene in spintronics, optoelectronic devices and biosensors. Man and colleagues studied advanced methods for fabricating 2D silicene/silicon structures, including de-alloying, metal reduction and topochemical techniques [35]. Their findings highlighted both the strengths and limitations of these fabrication methods, while also suggesting various applications for these materials. Further studies on silicene/silicon derivatives demonstrated their potential in energy storage, catalysis, biomedicine, electronics and sensor technologies. A comprehensive review of silicene’s properties, spanning theoretical and experimental studies, was carried out by Shan and co-workers [36]. The review covered the mechanical, electronic and spintronic properties of silicene, followed by a discussion of experimental work on the formation of silicene sheets on Ag(111) and other substrates. These studies provided key insights into the phase behaviour and electronic characteristics of silicene on metallic surfaces. Additionally, the team summarized the potential applications of silicene in sensors, nanoelectronics, quantum technology, electrode materials, and energy storage systems.

In addition to monolayer variants, 1D structures such as silicene nanoribbons garner substantial interest [37–39]. These structures, which confine electrons in two dimensions while enabling unimpeded motion in a single direction, engender a gamut of novel properties that accentuate the prospective applications of silicene. The objective of this study is to investigate a novel structural variant of silicene nanoribbons, specifically AlSi nanoribbons (AlSiNRs), characterized by a 1D structure with a width of five atoms and hydrogen-functionalized edges. Each unit cell of the AlSiNR consists of seven Si atoms, three Al atoms and four hydrogen atoms functionalized along the two edges. Utilizing DFT, this study aims to comprehensively examine the electromagnetic and optical properties of pristine AlSiNRs, as well as the adsorption behaviour of CO and H_2_S molecules. The primary goal is to elucidate the potential applications of AlSiNRs, particularly in the design of specialized gas sensors for CO and H_2_S detection, thus highlighting the material’s prospects in sensor technology.

## 2. Methods

The present study employs DFT as its theoretical framework, with all simulations conducted using the Vienna Ab initio Simulation Package software. The calculations are based on the generalized gradient approximation using the Perdew–Burke–Ernzerhof exchange-correlation functional and corresponding pseudopotentials.

For electronic structure calculations, the self-consistent field (SCF) approach is utilized to achieve electronic convergence. The convergence of the total energy is controlled by the EDIFF parameter, which is set to 1 × 10^−6^ eV, ensuring that the difference in total energy between successive iterations is below this threshold. This guarantees that the charge density reaches consistency with the potential generated within the system. The maximum number of SCF iterations allowed per step is controlled by the NELM parameter, which is set to its default value of 100, though convergence is typically reached well within this limit.

The ALGO parameter is set to Fast, applying a hybrid Davidson and RMM-DIIS algorithm to speed up the convergence of the electronic minimization. Charge density mixing parameters, AMIX = 0.2 and BMIX = 0.0001, are employed to stabilize convergence. Additionally, the EDIFFG parameter, set to −0.01 eV Å^−1^, serves as the criterion for force convergence, ensuring that atomic forces on each ion are minimized during structural relaxations. The k-point sampling in the Brillouin zone is controlled by the KPOINTS grid. For structural relaxation, a coarse grid of 1 × 1 × 11 is initially applied. After relaxation, a denser grid of 1 × 1 × 100 is used to compute electronic properties such as the band structure and density of states, providing higher accuracy in the sampling of electronic states. All these parameters are carefully chosen to balance computational efficiency with accuracy, ensuring the reliability of the calculated electronic, structural, and energetic properties.

The adsorption energy of various configurations is determined employing the following formula [40]:


(2.1) $$E_{a}=E_{t}-E_{p}-E_{CO/H_{2}S},$$

where *E*_a_ is the adsorption energy, *E*_t_ is the total energy of adsorbed configurations, *E*_p_ is the total energy of pristine configuration and $E_{CO/H_{2}S}$ is the energy of CO/H_2_S gas molecules.

The dielectric function is as follows:

(2.2) $$\varepsilon =\varepsilon_{re}\left(\omega \right)+\varepsilon_{im}\left(\omega \right),$$

where $\varepsilon_{re}\left(\omega \right)$ and $\varepsilon_{im}\left(\omega \right)$ are the real and imaginary parts of the dielectric function, respectively.

## 3. Structural properties and electromagnetic properties

**Figure 1.**
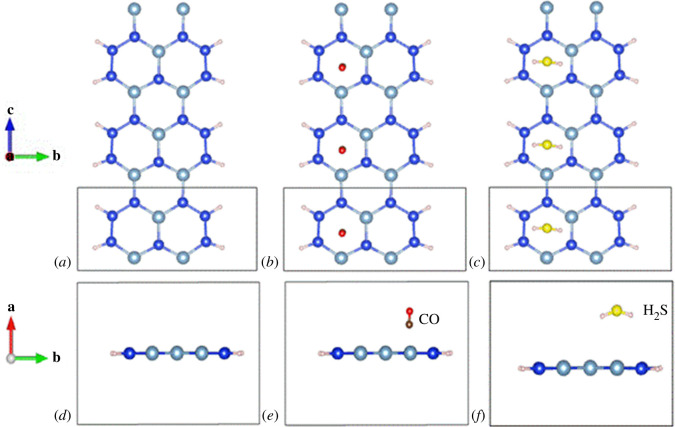
Top view and side view of configurations. Pristine AlSiNRs (*a,d*), CO-adsorbed AlSiNRs (*b,e*), H_2_S-adsorbed AlSiNRs (*c,f*) (the elements Si, Al, C, O and S are blue, silver white, copper, red and yellow, respectively, with H atoms on both edges).

Figure 1 depicts the top and side views of the structural configurations under investigation in this study. Examination of the pristine configuration (figure 1*a*,*d*) reveals its relatively planar nature, corroborated by table 1, which indicates an average angle of 119.998° between adjacent bonds—consistent with the expected angle of 120° in a flat hexagonal honeycomb structure. However, the hexagonal structure’s angles are not uniform, with the largest angle measuring 123.411° and the smallest angle 117.556° (table 1). Figure 1*b*,*c,e*,*f* illustrates the doping configurations adsorbing CO and H_2_S. Table 1 provides the parameters of these adsorption configurations, revealing a similarity in the angle between adjacent bonds in both adsorbed configurations, measuring 119.999°. This suggests that secondary to adsorption, the structure tends towards greater planarity. Notably, the H_2_S adsorption configuration exhibits the most significant deviation, with the largest and smallest angles between adjacent bonds measuring 154.309° and 67.589°, respectively. The substantial disparity between *α*_max_ and *α*_min_ signifies considerable alteration in bond lengths following H_2_S adsorption.

**Table 1.** Structural parameters of the configurations (*d*_1_ and *d*_2_ are the bond lengths of Si–Si and Si–Al, respectively; *α* is the angle between two adjacent bonds, *E*_a_ is the adsorption energy, *μ* is the total magnetic moment in a unit cell).

configurations	*d*_1_ (Å)	*d*_2_ (Å)	*α* (°)	*α*_max_ (°)	*α*_min_ (°)	*E*_a_ (eV)	*μ* (μ_B_)
pristine	2.237	2.408	119.998	123.411	117.556	—	0.98
CO-adsorbed AlSiNRs	2.454	3.034	119.999	140.551	107.824	−3.353	1.24
H_2_S-adsorbed AlSiNRs	2.846	3.714	119.999	154.309	67.589	−0.575	0.55

Specifically, table 1 delineates the Si–Si bond lengths in the pristine, CO-adsorbed and H_2_S-adsorbed configurations as 2.237, 2.454 and 2.846 Å, respectively—evidencing a notable increase post-adsorption. The Si–Si bond is weakened subsequent to CO/H_2_S adsorption, with a more pronounced effect observed following H_2_S adsorption. Furthermore, the bond length between Si and Al similarly increases post-CO/H_2_S adsorption, with increments of 0.626 and 1.306 Å, respectively, indicating a weakening of the Si–Al bond following gas adsorption. When compared with the bond lengths in silicene nanoribbons (SiNRs) (2.25 Å) [41], silicon carbide nanoribbons (SiCNRs) (1.77 Å) [37], germanene nanoribbons (GeNRs) (2.45 Å) [42] and graphene nanoribbons (GNRs) (1.42 Å) [43], the results reveal that the Si–Si bond length in the AlSiNR structure is similar to that in SiNRs, shorter than in GeNRs, but longer than in both SiCNRs and GNRs.

Table 1 also presents the adsorption energy of the doping configurations, with both CO and H_2_S displaying negative values, indicative of energetically favourable adsorption processes and structural stability. Notably, the CO adsorption process exhibits stronger stability, as evidenced by its lower adsorption energy (−3.353 eV). Another noteworthy parameter is the total magnetic moment within a unit cell (*μ*). Table 1 reveals that the pristine configuration possesses magnetic properties, with a total magnetic moment of 0.98 μ_B_. Subsequent to CO adsorption, an increase in magnetism is observed (*μ* = 1.24 μ_B_), while H_2_S adsorption induces a reduction in magnetism (*μ* = 0.55 μ_B_), illustrating the potential for magnetism control via CO/H_2_S gas adsorption. In comparison with the studied SiNR structure, which is a semiconductor with an energy gap of 0.39 eV and exhibits no magnetic properties [41], it can be concluded that the fundamental distinction between AlSiNRs and SiNRs arises from the presence of Al atoms in the structure.

**Figure 2.**
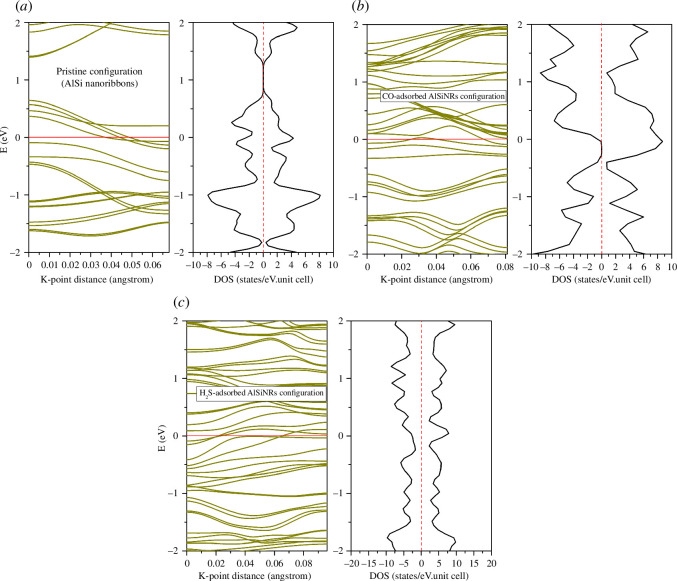
Band structure and density of states of configurations: (*a*) pristine, (*b*) CO-adsorbed AlSiNRs, (*c*) H_2_S-adsorbed AlSiNRs.

Figure 2 illustrates the energy band structure and density of states for the examined configurations. Evidently, all three configurations manifest metallic behaviour. The presence of magnetism is discernible through the asymmetry observed between spin-up and spin-down states. Notably, a marked augmentation in the density of states is observed when contrasting the pristine configuration with the CO/H_2_S adsorption configurations, particularly within the conduction band. The doping profiles exhibit multiple peaks within the conduction band and proximal to the zero-energy level.

**Figure 3.**
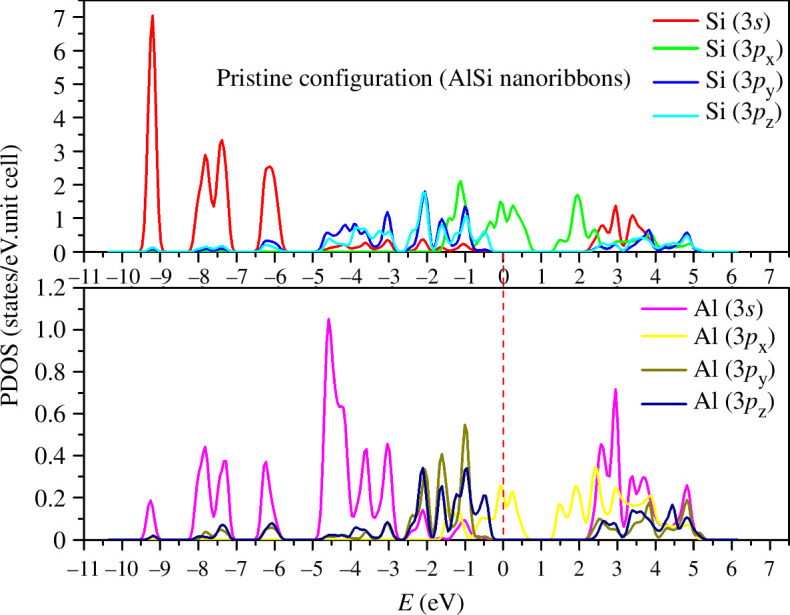
Partial density of states (PDOS) in pristine configuration.

The partial density of states (PDOS) for the pristine configuration is depicted in figure 3. In the case of silicon (Si), the Si(3*s*) states predominantly occupy the deep energy region, with minimal representation at the upper extremities of the valence region and no presence in the lower portion of the conduction band. The peak intensity of Si(3*s*) is observed at an energy level of 9.25 eV. Conversely, the 3(*p*_*x*,*y*,*z*_) states of Si are concentrated within the lower energy spectrum, with 3(*p*_*x*_) states primarily clustered around the zero-energy level. Analysing the PDOS of aluminium (Al) reveals a contrasting pattern, with Al(3*s*) states exhibiting greater prominence within the low-energy region, attaining their highest peak at 4.5 eV. Similar to Si, Al(3*p*_*x*_) states are predominantly concentrated near the zero-energy level. Complex multi-orbital hybridizations spanning various energy ranges serve as the foundation for σ and π bonding interactions between atoms, notably exemplified by Si(3*s*, 3*p*_*x*,*y*,*z*_)–Al(3*s*, 3*p*_*x*,*y*,*z*_) multi-orbital hybrids across multiple energy intervals such as from −9.5 to −9 eV, from −8.25 to −7 eV, from −6.5 to −5.6 eV and from −5 to −3.2 eV.

**Figure 4.**
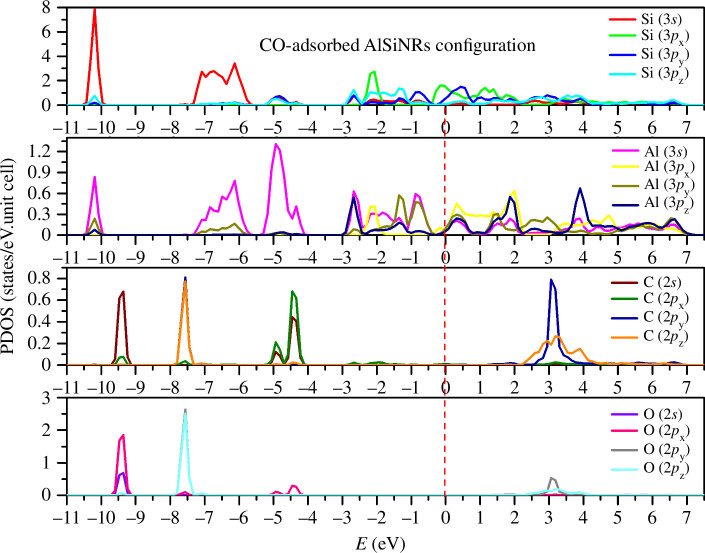
Partial density of states (PDOS) in CO-adsorbed configuration.

Subsequent analysis delves into the disparity of PDOS within CO and H_2_S gas adsorption configurations. Initially, examining the CO adsorption configuration (figure 4), discernible shifts in the largest peaks of Si(3*s*) and Al(3*s*) towards deeper energy levels are observed (10.25 and 4.8 eV, respectively). Moreover, the emergence of (*s,p*) states around the Fermi level results in a denser density of states compared to the pristine configuration. In contrast, the presence of C(2*s*, 2*p*_*x*,*y*,*z*_) and O(2*s*, 2*p*_*x*,*y*,*z*_) states within the low-energy spectrum is minimal. Clear multi-orbital hybridizations between carbon (C) and oxygen (O) are apparent across all energy ranges, while hybridization with Si and Al is evident primarily within energy ranges from −5 to −4 eV. Consequently, CO adsorption amplifies the density of states at the apex of the valence band and the nadir of the conduction band, thereby augmenting the metallic properties of the adsorbed system relative to its pristine counterpart.

**Figure 5.**
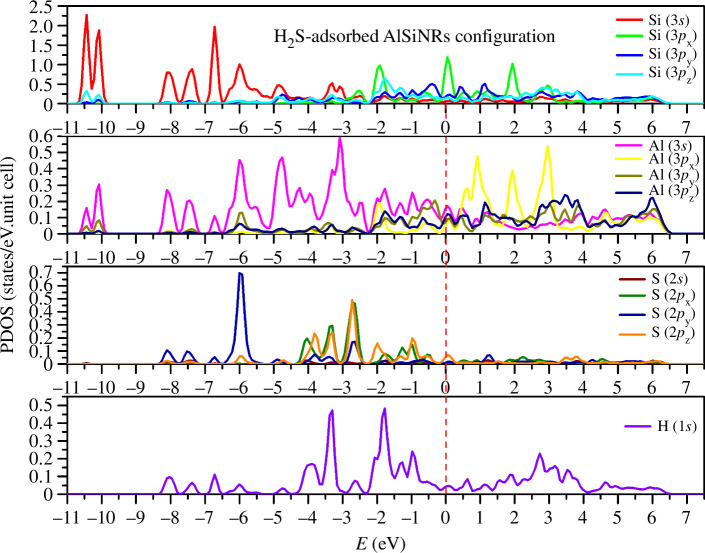
Partial density of states (PDOS) in H_2_S-adsorbed configuration.

In the case of the H_2_S adsorption configuration (figure 5), a notable shift is observed in the largest peak of Si(3*s*) towards a lower energy level (−10.5 eV), while the highest peak of Al(3*s*) converges closer to the zero-energy level at −3.1 eV. Analogous to CO adsorption, the states become more concentrated around the Fermi level following H_2_S adsorption, thereby enhancing the metallic character of the adsorbed system. Hybridizations between S(3*s*, 3*p*_*x*,*y*,*z*_) and Al/Si(3*s*, 3*p*_*x*,*y*,*z*_) occur across multiple energy ranges, exhibiting a more intricate profile compared to the hybridization between C/O and Al/Si.

**Figure 6.**
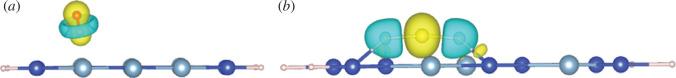
Charge density difference in configurations: (*a*) CO-adsorbed AlSiNRs, (*b*) H_2_S-adsorbed AlSiNRs (the blue area represents charge concentration, and the yellow area represents charge reduction).

The charge density difference is visually examined via figure 6. Redistribution of charge density is observed surrounding the adsorbed gas atoms, whereas the extent of charge transfer between CO/H_2_S and AlSiNRs is found to be negligible. Specifically, a transfer of charge towards the outer regions of the carbon (C) and oxygen (O) atoms is evident, whereas a transfer of charge from sulfur (S) to hydrogen (H) is observed.

## 4. Optical properties

**Figure 7.**
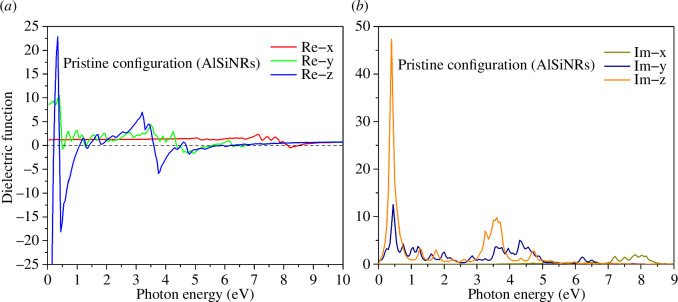
The (*a*) real and (*b*) imaginary parts of the dielectric function of the pristine configuration.

The optical properties of materials are delineated by the dielectric function, which comprehensively elucidates the propagation characteristics of radiation within the material. Additional optical parameters such as refractive index, absorption coefficient, and others can be derived from the dielectric function. Figure 7 portrays the dielectric function of the pristine AlSiNR configuration, comprising both its real and imaginary components. The real part, depicted in figure 7*a*, signifies the degree of retardation experienced by radiation traversing the material. Notably, the prominent peaks in both the 0*y* and 0*z* directions reside within the low-energy spectrum (approximately 0.4 and 0.35 eV, respectively), corresponding to the infrared radiation regime. This observation is explicable due to the limited energy of infrared radiation, impeding its penetration through the material. Moreover, the 0*z* direction, characterized by a denser atomic arrangement, imposes greater electromagnetic wave obstruction, as evidenced by the largest peak. Conversely, the 0*x* direction, being atomically thin, facilitates relatively unhindered propagation of electromagnetic waves. Notably, the occurrence of negative dielectric constant values within certain energy ranges denotes superconductivity, indicating minimal energy dissipation during radiation propagation within the material.

The imaginary part of the dielectric function delineates the gradual attenuation of radiation within the material. As depicted in figure 7*b*, the primary peaks in the 0*z* and 0*y* directions are confined to the infrared energy range, aligning with the aforementioned analysis in figure 7*a*, where infrared radiation undergoes rapid attenuation along the 0*y* and 0*z* directions. In contrast, within the 0*x* direction, damping of radiation is evident only within the energy range spanning from 7 to 8.5 eV, indicating comparatively modest attenuation.

**Figure 8.**
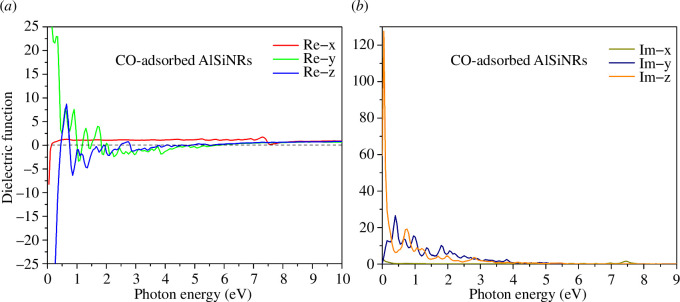
The (*a*) real and (*b*) imaginary parts of the dielectric function of CO-adsorbed configuration.

In the CO gas adsorption configuration (see figure 8), akin to the pristine state, the predominant peaks of the real component of the dielectric function reside within the infrared spectrum. However, the introduction of CO induces a notable retardation of radiation propagation along the 0*y* direction, contrasting with enhanced propagation along the 0*z* direction compared to the pristine configuration. Analogous to the pristine state, the presence of numerous negative dielectric constant values renders the material conducive to efficient radiation transmission with minimal energy dissipation.

**Figure 9.**
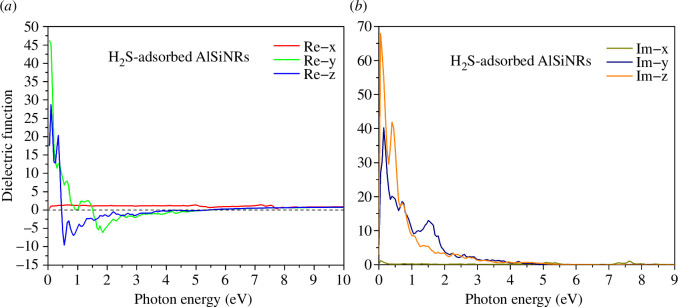
The (*a*) real and (*b*) imaginary parts of the dielectric function of H_2_S-adsorbed configuration.

Following CO adsorption, a substantial augmentation in the peaks of the imaginary component of the dielectric function is observed in both the 0*y* and 0*z* directions, indicating accelerated radiation attenuation upon CO introduction. Noteworthy alterations in the dielectric function manifest in the H_2_S gas adsorption configuration (see figure 9), particularly with the largest peak of the real component along the 0*z* axis shifting towards a lower energy range. Furthermore, for electric wave energies exceeding 3 eV, a marked reduction in the material’s electromagnetic wave resistance is evident.

The H_2_S adsorption configuration also exhibits expedited radiation attenuation, as evidenced by the heightened peaks of the imaginary component of the dielectric function (see figure 9*b*). Notably, the largest peak in the 0*y* direction attains a value of 40, surpassing those of the CO adsorption and pristine configurations, which register values of 26 and 12.5, respectively. Collectively, these configurations underscore the propensity for easier electromagnetic wave propagation within the material at wavelengths shorter than hose of ultraviolet radiation.

**Figure 10.**
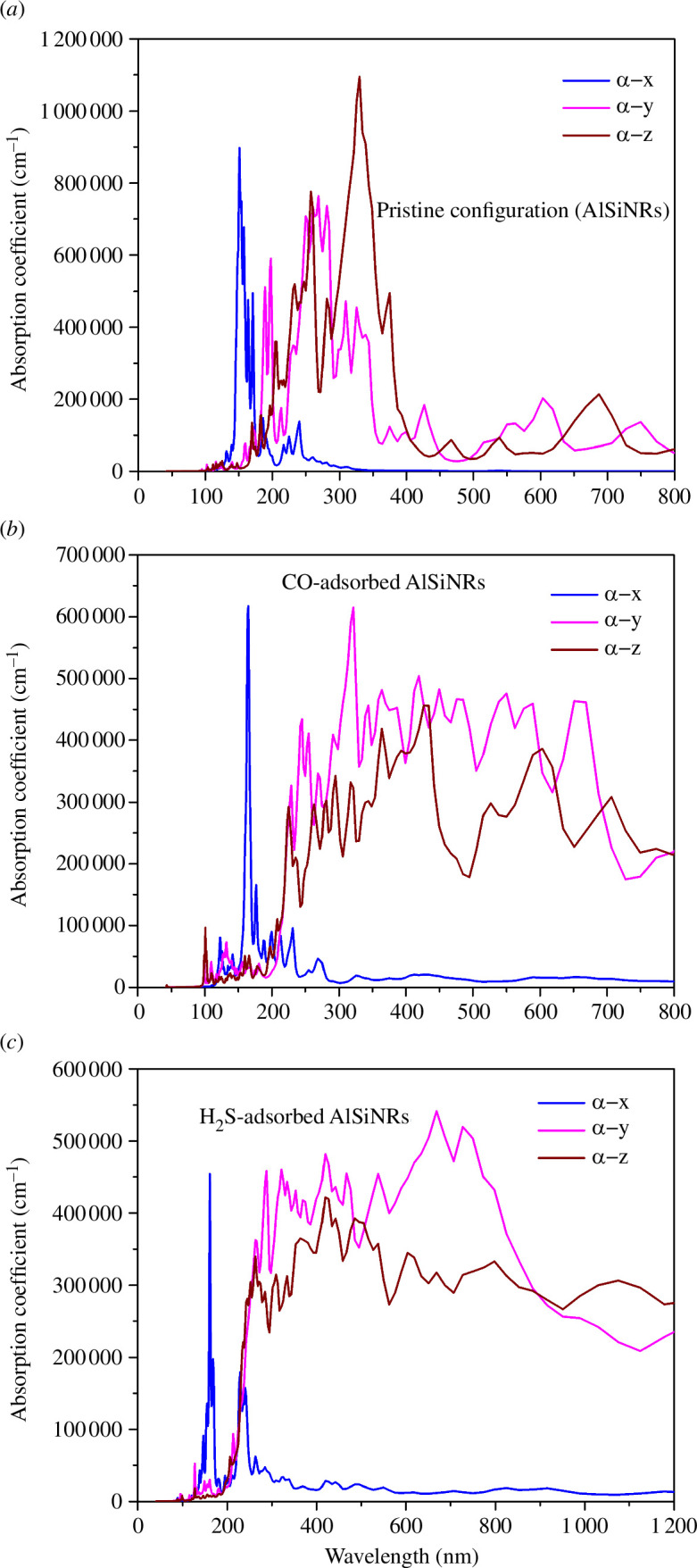
(*a*) The absorption coefficient for pristine configuration. (*b*) The absorption coefficient for CO-adsorbed configuration. (*c*) The absorption coefficient for H_2_S-adsorbed configuration.

The absorption coefficient serves as a crucial metric elucidating the intricacies of electromagnetic wave propagation within the material. It quantifies the energy dissipation occurring as electromagnetic waves traverse a unit thickness of the material (refer to figure 10). Across all three configurations, a salient observation emerges: for radiation with wavelengths below 100 nm, the absorption coefficient registers at 0, indicative of optical transparency within the material. Furthermore, a conspicuous disparity in absorption coefficients along the 0*x*, 0*y* and 0*z* directions manifests for electromagnetic wavelengths exceeding 100 nm, underscoring the material’s optical asymmetry.

Delving deeper into each configuration unveils discernible disparities following gas adsorption. In the pristine state, the apex of the absorption coefficient resides along the 0*z* direction, peaking at a wavelength of 330 nm. Conversely, upon CO and H_2_S gas adsorption, the apex shifts to the 0*y* direction, with peak wavelengths measuring 320 and 670 nm, respectively. Notably, beyond a 400 nm wavelength, the absorption coefficient precipitously declines in the pristine configuration, whereas in the gas adsorption configurations, it sustains elevated levels. This phenomenon signifies that upon CO/H_2_S gas adsorption, the material exhibits enhanced photon absorption capability, facilitating electron excitation to higher energy states, thus accentuating deviations from the original configuration.

**Figure 11.**
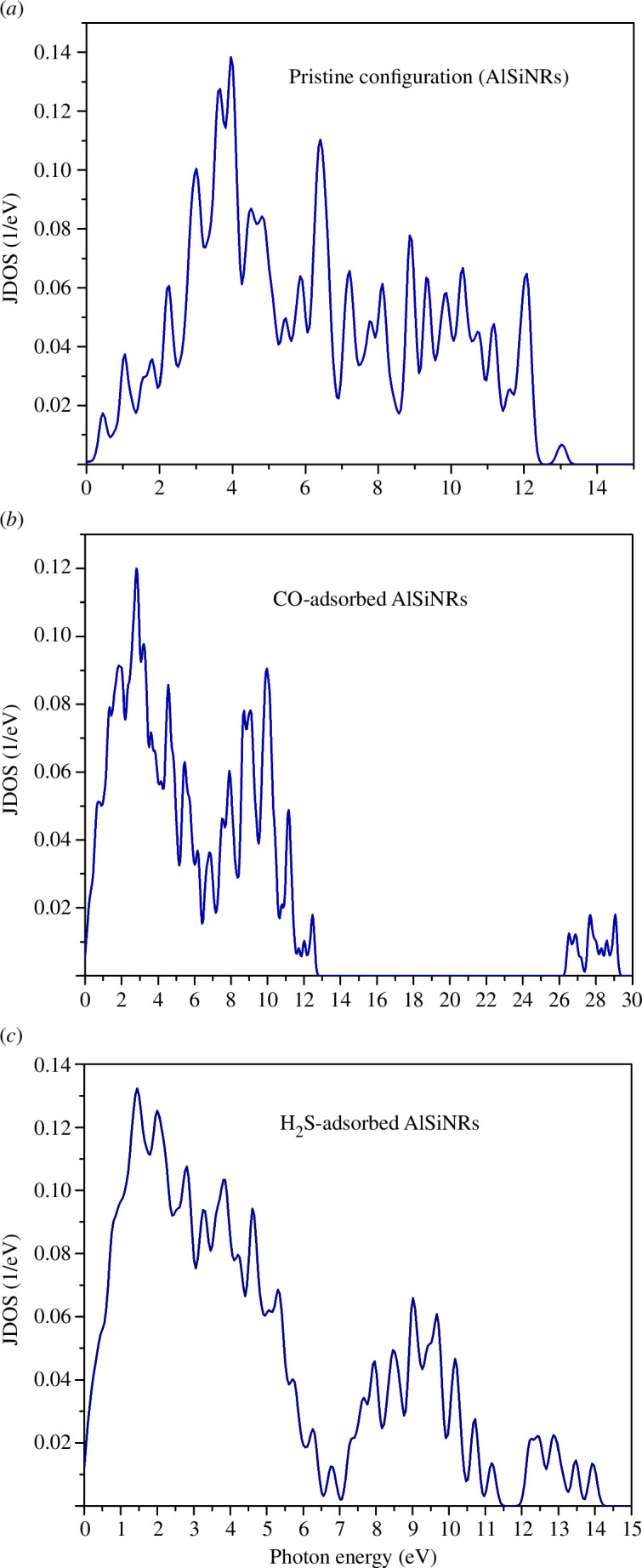
(*a*) The total joint density of states (JDOS) of pristine configuration. (*b*) The total JDOS of CO-adsorbed configuration. (*c*) The total JDOS of H_2_S-adsorbed configuration.

The total joint density of states (JDOS) quantifies the density of electron–hole pairs per unit of energy upon excitation of the system by an electromagnetic wave (see figure 11). Notably, even at relatively low photon energy levels, the system generates electron–hole pairs, a phenomenon congruent with the metallic energy band structures depicted in figure 2. The primary peak of JDOS corresponds to the pristine configuration, CO adsorption, and H_2_S adsorption, occurring at energy levels of 4, 2.5 and 1.6 eV, respectively.

In the pristine configuration, electron–hole pair generation ceases when photon energy surpasses 13.2 eV, consistent with the theoretical framework stipulating selective electron state transitions. However, a noteworthy deviation arises in the CO adsorption configuration, where electron–hole pair generation occurs within the energy range of 26 to 29 eV, a phenomenon absent in other configurations.

**Figure 12.**
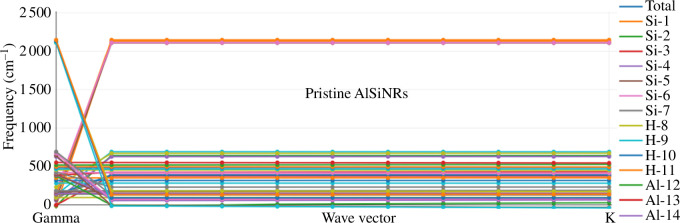
Phonon dispersion of pristine configuration (AlSiNRs).

The results of the phonon dispersion calculations for the pristine AlSiNR configuration are presented in figure 12. The absence of any imaginary (negative) frequencies in the phonon spectrum confirms the dynamical stability of the AlSiNR structure. In certain phonon branches, negative slopes are observed at low values of the K wave vector, indicating negative group velocities, which are characteristic of acoustic metamaterials. As the K wave vector increases, the slopes of these phonon branches approach zero, suggesting that the material exhibits low thermal conductivity.

## 5. Conclusions

This study highlights the electromagnetic and optical properties of CO and H_2_S adsorption on AlSiNRs. Computational results reveal that the pristine and gas-adsorbed systems exhibit magnetic metallic behaviour, with CO adsorption enhancing magnetism and H_2_S reducing it. This suggests the potential for tuning the magnetic properties of AlSiNRs for gas-specific sensing applications. PDOS analysis reveals complex orbital hybridizations that contribute to the formation of stable σ and π bonds, maintaining the hexagonal structure of AlSiNRs. Despite minimal overall charge transfer between the adsorbed gases and the substrate, localized charge redistribution occurs between atoms (C–O for CO and S–H for H_2_S), which is critical for understanding the system’s electronic behaviour.

Optical properties were thoroughly explored, showing that the system becomes transparent to light at photon energies above 12.42 eV, with notable optical asymmetry below this threshold. This behaviour could be advantageous for optical sensing devices. Additionally, electron–hole pairs are generated selectively at specific energy ranges, highlighting the potential for tuning the material’s optical response based on incident photon energy.

The study concludes that AlSiNRs show strong potential for gas sensing, particularly in CO detection due to its stronger interaction with the surface. Future work could expand on these findings by exploring additional gases and incorporating experimental validation to further demonstrate the practical applications of AlSiNRs in sensor technologies.

## Data Availability

Data are available from the Dryad Digital Repository [44].
